# Rush Medical College

**Published:** 1876-01

**Authors:** Walter Hay

**Affiliations:** Adjunct Prof. of Theory and Practice of Medicine, Lecturer, and Clinical Lecturer on Nervous and Mental Diseases


### Clinics.

### RUSH MEDICAL COLLEGE.

### NEUROLOGICAL CLINIC.

BY WALTER HAY, ADJUNCT PROF. OF THEORY AND PRACTICE OF MEDICINE, LECTURER, AND CLINICAL LECTURER ON NERVOUS AND MENTAL DISEASES.

(Reported by EDGAR SNYDER, Clinical Assistant.)

### LOCOMOTOR ATAXIA.

*Gentlemen*—The patient, William Tonks, whom I here present to you, an Englishman, thirty-six years of age, is, and has always been moral and temperate in his habits, as his appearance indicates. His trade is that of a brass turner, which he has prosecuted continuously for seven years, maintaining a standing position constantly during ten hours daily.

He was sent to this clinic by Prof. Edwin Powell, on the 5th of Sept., 1875, giving the following history of his case:

In 1871 he began to feel low spirited and nervous, with constant dread of falling, which he attributed to weakness.

In 1873 he began to feel pains in the back of the neck and back of the hip joints, shooting downward toward the feet, and soon afterward a sensation as if he had been sitting a long time and his legs had gone to sleep.

He soon found it difficult to keep step with his companions in walking, feeling obliged to occupy his mind with his walking to watch his feet, which were always getting too far ahead of him. He would also lose his balance when stooping to wash his face. His bowels have been habitually constipated, and his urine is evacuated slowly.

His head has troubled him greatly, not so much from pain as with a dull, heavy feeling; he is easily excited, both mentally and physically, and as easily exhausted, especially in hot weather, and finds his mind much occupied with his feelings.

The pains in his lower extremities are less severe when he lies down than when he occupies any other position, but always recur within five minutes after he gets up in the morning.

He has been under various modes of treatment for various imaginary maladies, *i. e.*, for spermatorrhœa, for paralysis, etc., having taken a long course of "electric baths," but has gradually grown worse. To the latter treatment he is disposed to attribute an exaggeration of his dorsal pains, with how much reason it is difficult to say.

Having heard the history of this case, we will proceed to examine its objective features, as they present themselves to us. First, as regards intellection, the patient's expression, bearing, and conversation, enable us to conclude positively the entire exemption of his intellectual faculties from disease. His special sensation, moreover, is perfect; sight, hearing, taste and smell being all quite normal in their manifestations.

As he has been treated for paralysis, and pronounced a paralytic by medical authority, let us see upon what foundation such a diagnosis could rest. We will therefore examine into the function of motility. You perceive that the muscles of his face contract in perfect subordination to the will and the emotions, and that his upper extremities have lost none of their facility of movement nor muscular strength, the grasp of his hand being strong and vigorous.

We will now ask the patient to walk, and perceive that he does so with difficulty, his feet striking the ground outside of the line of gravitation, having been first thrown forward through an arc longer than the required length of the step, and falling backward—illustrating the patient's description of his feet getting ahead of him—strike the ground suddenly and abruptly, first with the heel, then with the sole of the foot, making two distinct sounds, the first a blow, the second a slapping sound the distinctive characters of these two sounds being clearly perceptible to the ear with each successive step.

His motion is a combined roll and plunge, and he keeps his eyes closely fixed upon his feet.

We will now place him in a standing position, and we perceive that his feet are placed much farther apart than is usual or natural, and when we make him approximate the inner edges of his feet, his position becomes unsteady, and when he raises his head or shuts his eyes, he topples over and loses his balance like an inverted pyramid.

Is he paralyzed in his lower extremities? We will now make an *experimentum crucis.* Seated upon the floor, and crossing his legs under him, the outer sides of his feet and legs resting upon the floor, he folds his arms and raises himself to a standing position by the action of the muscles of the legs alone, an experiment which very few men in perfect health could accomplish successfully. The theory of paralysis must be absolutely excluded.

With reference to sensibility, we have heard him speak of pains in his back, in his hips and legs, shooting downward to his feet, and of numbness of his lower extremities, "as if his feet and legs had gone to sleep." He tells us, moreover, that he feels as if there were something soft under his feet; that he feels inequalities in the ground with difficulty.

If we examine the soles of this man's boots we perceive that the heels are quite worn away at their outer and hinder edge, whereas the toes show no signs of wear. This is the result of the peculiar gait, and is so remarkable, that one might almost make the differential diagnosis between this and some other forms of disease by an inspection of the patient's boots alone. Upon removing his boots and socks, we touch the soles of the feet with the pencil, drawing the point quickly along the surface, but he perceives no sensation.

The instrument which I hold in my hand is an æsthesiometer, of which there are several forms devised by different neurologists, this one being that known as Hammond's, having been designed by Dr. W. A. Hammond, of New York, and consists of a small pair of dividers having a transverse arm graduated. By means of this instrument the index of sensibility can be measured. This index of sensibility is the distance at which two points in contact with the skin may be distinguished; of course it varies with the locality. By applying this instrument to the soles of this man's feet, we find that the points can be distinguished as two, at distances varying from one-half to three-quarters of an inch upon different portions of the surface; at shorter distances apart, he is able to distinguish but one point, and that only when quite a deep impression is made upon the skin.

Here, then, are the first positive, objective evidences of perverted sensibility which we have yet detected: diminished sensibility—dysæsthesia—and diminished reflex irritability, exhibited by the insensibility of the sole of the foot to the point of the pencil drawn rapidly across it, which, under ordinary circumstances, would have excited the sensation of tickling, and occasioned a jerking away of the feet from contact with the object. I said the first positive, objective evidences of perverted sensibility, because pain and numbness are subjective purely, and we are entirely dependent for our knowledge in these particulars, upon the feelings of the patient, often very unreliable guides.

You will perhaps ask how these derangements of sensibility, dysæsthesia, and diminished reflex irritability, can alone produce the disturbances of motility exhibited in this patient. If you will reflect upon the intimate and necessary relation existing between sensation and motion in the acts of locomotion, the mystery will disappear. In the act of walking, the feet are moved by muscular action, but are guided to their proper positions by the sense of touch, stimulated by contact of the surface of the ground and the sole of the foot. It is by the sense of touch that one is enabled to adapt the steps to the inequalities of the surface over which he walks, and his progression, when once he acquires the art of walking, is accomplished rather by reflex than by distinctly voluntary efforts. Thus the impression made upon the nerves of the sole of the foot by the surface upon which it rests, is transmitted along sensory nerves through the posterior roots of the spinal nerves to the posterior cornua of gray matter in the cord, and is here converted by cell action into a motor impulse, in obedience to which when transmitted to the muscles, motions are performed. If the stimulus be insufficient, and the influence of the will is required to complete the motion, the impression is transmitted upwards to the centres of consciousness and volition. Now, in the various locomotory actions, numerous muscles and groups of muscles are called into play, each bearing its proportionate share of the labor, in order that symmetry of motion may be attained.

If, therefore, there be any obstruction to or interference with the perfect transmission of every impression through the posterior gray substance of the cord upward, there must result more or less incoördination of muscular action, by means of which locomotion, and the maintenance of equilibrium even, become seriously impaired.

The patient has told us, moreover, that his bowels are constipated habitually, and his urine evacuated slowly and with an effort. The functions of these viscera are mainly reflex, or, at most, consensual, and would be rendered less active by any cause which would induce dysæsthesia or anæsthesia of their mucous linings, thereby rendering them less sensitive to the presence of their contents, and less responsive to reflect the influence of their impressions.

You have observed that the patient, in walking, kept his eyes fixed upon his feet, and lost his balance when they were closed or averted. This is done in obedience to the necessity felt to supplement the loss of the guidance of the touch by the sight, when the communication between the sensitive surface and the nerve centres is intercepted.

Other portions of the nervous system being excluded by the negative evidence, we are led by induction to the posterior gray substance of the spinal cord as the seat of the morbid process, and to the conductors of sensory impressions as the diseased tracts.

Of the nature of the morbid process we may learn something from the history of the case. The man's occupation has required him to maintain a standing position uninterruptedly for ten hours daily during a number of years, entailing great muscular endurance and extraordinary drafts upon the spinal cord for nerve power to maintain that endurance. For the generation of this great amount of nerve force, a large blood supply was demanded, and we have hyperæmia; but this blood supply is the source of nutriment to all the tissues of the cord alike in their due proportions; now there are not only nerve cells and nerve fibres in the spinal cord, but there is connective tissue likewise, the neuroglia, the cement or matrix, in which these nerve cells and fibres are imbedded; and while there is a due proportion in the supply of nutriment to each of these, there is a disproportion in the waste, nerve cells and fibres sustaining retrograde metamorphosis under their condition of increased activity, to a much greater extent than the neuroglia, which therefore becomes the seat of a hyperplasia.

This view of the pathology of locomotor ataxia is sustained by post-mortem examination, in which the neuroglia is found indurated in scales and flakes, either aggregated, (*sclérose diffuse* of Duchenne,) scattered throughout the continuity of the cord, (*sclérose disseminée,*) or encircling the cord, (*sclérose corticale.*)

I shall detain you but a few moments while I indicate the best treatment for this condition, and in this, as in all other therapeutic theories, we must keep in mind the old maxim, *remove the cause.* Now, what was the ultimate cause, the primum mobile, of this long chain of morbid processes? Without doubt, long continued muscular effort. We will therefore remove the cause, and to protect him from the ill consequences of muscular exertion, which has proven so disastrous to him, will direct him to remain in bed, to rest continuously.

But we may do more than this; we can diminish the blood supply to the cord, and to this end we will direct him to take one-third of a grain of nitrate of silver three times daily, and will, as the case progresses, substitute for it the fluid extract of secale cornutum.

Nov. 6th. The patient says he is much better; is quite hopeful, and his walk bears visible testimony to his improvement.

